# Plasma Proteomic Signatures of Pediatric Sepsis Reveal Persistent Inflammation and Phase‐Specific Biomarkers

**DOI:** 10.1096/fba.2026-00006

**Published:** 2026-03-09

**Authors:** Fahd Alhamdan, Yi‐Cheng Sin, Erik Malm, Hanna Van Pelt, Samuel Kim, LeeAnn Higgins, Yue Chen, Koichi Yuki

**Affiliations:** ^1^ Department of Anesthesiology, Critical Care and Pain Medicine, Cardiac Anesthesia Division Boston Children's Hospital Boston Massachusetts USA; ^2^ Department of Immunology Anaesthesia Harvard Medical School Boston Massachusetts USA; ^3^ Broad Institute of MIT and Harvard Cambridge Massachusetts USA; ^4^ Department of Biochemistry, Molecular Biology and Biophysics University of Minnesota Minneapolis Minnesota USA

**Keywords:** pediatric, proteomics, sepsis, sterile inflammation

## Abstract

Sepsis remains a leading cause of pediatric morbidity and mortality, yet its molecular underpinnings are poorly understood. Here, we performed mass spectrometry–based plasma proteomics and cytokine profiling in pediatric sepsis patients at the acute phase (AP) and recovery phase (RP), alongside preoperative surgical controls. In AP vs. control, we identified 41 differentially abundant (DA) proteins, including acute‐phase reactants and complement factors, with persistent but attenuated expression in RP. Pathway analysis revealed sustained enrichment in inflammatory and complement activation processes during both AP and RP, with partial restoration of immune surveillance and vascular homeostasis in recovery. Machine learning highlighted complement components (C9, C1R) and LRG1 as candidate AP biomarkers, and S100A9 as an RP‐associated marker. Comparative analysis with adult sepsis proteomes uncovered age‐specific complement activation patterns: adults displayed higher classical pathway activity, whereas pediatric patients exhibited enhanced alternative pathway activity. Cytokine profiling confirmed sustained immune activation and endothelial perturbation across sepsis phases. We also compared the sepsis cohort with the sterile inflammation (SI) cohort, which revealed distinct adaptive immune enrichment in sepsis while innate immune predominance in SI, enabling the identification of potential sepsis‐specific protein signatures. Together, these findings delineate the dynamic immune and vascular proteomic landscape of pediatric sepsis, reveal biomarkers distinguishing sepsis from sterile inflammation, and highlight age‐related complement pathway differences with potential therapeutic implications.

**Trial Registration:** ClinicalTrials.gov: NCT04103268, NCT04299828

## Graphical abstract

We enrolled a pediatric sepsis cohort at acute and recovery phases for plasma proteomics analysis. Abundantly expressed proteins in sepsis were identified and subjected to machine learning. In addition, the data was compared to adult sepsis and sterile inflammation.

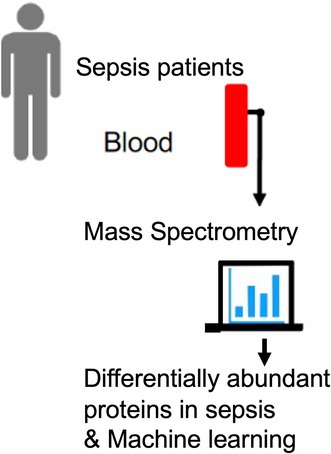

## 1 Introduction

Sepsis is a life‐threatening condition characterized by dysregulated host responses to infection, leading to life‐threatening organ dysfunction and high mortality rates worldwide [1, 2, 3]. In pediatric populations, the immunological landscape of sepsis is particularly complex due to the ongoing development of the immune system involving both innate and adaptive immunity, which can influence susceptibility, inflammatory trajectories, and recovery patterns [1, 4, 5]. The heterogeneity in pediatric immune responses, driven by factors such as age, comorbidities, and prior pathogen exposures, complicates both the clinical presentation and the development of universally applicable diagnostic and prognostic tools [6, 7].

Despite advances in antimicrobial therapy and supportive critical care, the early diagnosis, risk stratification, and individualized management of pediatric sepsis remain major challenges [8]. Current clinical scoring systems often rely on physiological and standard clinical laboratory parameters that may lag the underlying pathophysiological changes [9]. This gap underscores the urgent need for molecular biomarkers that not only facilitate timely diagnosis but also capture the evolving immunological state of patients across different stages of illness.

Proteomics offers a powerful, systems‐level approach to interrogate host responses by profiling the circulating collection of proteins, which reflects the complex interplay between inflammation, coagulation, endothelial function, and immune regulation [10, 11]. By capturing both acute‐phase reactants and short‐term homeostatic shifts, plasma proteomics can provide an insight into the persistence or resolution of pathogenic processes. While proteomic studies in adult sepsis have revealed signatures associated with mortality, immune suppression, and coagulation disturbances [12], pediatric sepsis remains underrepresented in large‐scale omics‐driven investigations, despite its distinct immunobiology [13]. While pediatric and adult sepsis difference is widely recognized including predisposing diseases, sites of infection, and immune system development [1], biological comparison has been limitedly performed.

Furthermore, a key clinical challenge lies in distinguishing sepsis from sterile inflammatory conditions, such as those triggered by trauma or surgery, which can present with overlapping clinical and laboratory features [14, 15]. Misclassification can delay appropriate antimicrobial treatment in infectious cases or lead to unnecessary antibiotic exposure in non‐infectious cases, contributing to antimicrobial resistance [16]. Molecular profiling approaches that can discriminate between infectious and sterile inflammation are therefore essential for guiding targeted therapy and improving patient outcomes.

We first performed comprehensive plasma proteomic and cytokine profiling in pediatric sepsis patients sampled during acute and recovery phases. Then, we conducted comparative analysis with adult sepsis proteomes. We also compared proteomics from patients experiencing sterile inflammation following cardiac surgery to characterize dynamic protein expression patterns and pathway perturbations across the sepsis phases. We revealed the dynamic immune and vascular proteomic landscape of pediatric sepsis, revealed biomarkers distinguishing sepsis from sterile inflammation, and highlighted age‐related complement pathway differences. The findings aim to advance the molecular understanding of pediatric sepsis and inform future diagnostic and therapeutic strategies.

## 2 Material and Methods

### 2.1 Human Sepsis and Control Subject Enrollment

The study was approved by the Institutional Review Board of Boston Children's Hospital, and written informed consent was obtained from parents or legal guardians; assent was obtained from patients when appropriate. Six pediatric sepsis patients at both the acute phase (AP) and recovery phase (RP), alongside five age‐matched preoperative surgical controls (Ctrl) were prospectively enrolled for study between May 2021 and January 2023. All procedures adhered to the Declaration of Helsinki, and the study was registered at ClinicalTrials.gov (NCT04103268). Eligible participants were aged 1 month to 18 years. Control subjects were otherwise healthy pediatric patients undergoing elective surgical procedures. Sepsis patients were enrolled based on a documented or suspected infection and pediatric Sequential Organ Failure Assessment (pSOFA) sub‐score ≥ 2 in at least one organ system at ICU admission [17]. Blood collected at this time point was defined as the acute phase. Patients were subsequently monitored during their ICU stay, with the recovery phase defined by a decrease in pSOFA scores from initial levels. Exclusion criteria included congenital heart disease, malignancy, autoimmune disorders, organ transplantation, human immunodeficiency virus (HIV) infection, or corticosteroid therapy. AP sample was collected at the time of ICU admission. RP sample was collected during the same ICU admission when pSOFA scores were reduced from the initial levels, usually 3–5 days after ICU admission. At each time point, 1 mL of peripheral blood was collected in heparin‐coated tubes and plasma was isolated by centrifuging blood for 10 min at 400 × *g*. They were aliquoted into multiple tubes to avoid freeze and thaw and stored at −80°C until use. They were subsequently subjected to proteomics and V‐plex analysis described below.

### 2.2 Human Sterile Inflammation (Post‐Surgery) Subject Enrollment

This single‐center prospective study was approved by the Institutional Review Board of Boston Children's Hospital. Neonates and infants scheduled to undergo congenital cardiac surgery with cardiopulmonary bypass (CPB) were eligible for inclusion. Exclusion criteria included the absence of CPB, preoperative active infection, chronic steroid therapy, history of malignancy, or the need for mechanical ventilation or inotropic support immediately prior to surgery. Patient enrollment occurred between May 31, 2022, and February 22, 2023. Detailed methodological procedures were previously described [18]. The study was registered at ClinicalTrials.gov (NCT04299828). Samples used in this study were collected on postoperative day 1.

### 2.3 Proteomic Sample Preparation

Proteomic sample preparation was performed as previously described by our group [19]. Briefly, plasma samples were briefly vortexed and denatured with two volumes of 9 M urea in 100 mM ammonium bicarbonate containing cOmplete protease inhibitor (Roche), followed by incubation at room temperature with rotation for 10 min. After incubation, samples were centrifuged to remove insoluble material. The supernatant was treated with 10 mM tris(2‐carboxyethyl)phosphine (TCEP) and 10 mM iodoacetamide (IAA) in 100 mM ammonium bicarbonate for protein reduction and alkylation and incubated with rotation in the dark for 30 min. The reaction was quenched with 15 mM cysteine. 50 μg of proteins in each sample were digested with trypsin at a 1:50 (w/w) enzyme‐to‐substrate ratio overnight at 37°C, followed by a second digestion at a 1:100 ratio for 4 h at 37°C. The reaction was terminated with 5% trifluoroacetic acid (TFA) prior to desalting.

### 2.4 Nano‐HPLC‐MS/MS Analysis

Digested peptides were desalted using C18 stage tips as previously described [19, 20]. Then 100 ng of the peptides was injected in a volume of 2 μL of loading solvent (98:2, water: acetonitrile, 0.1% formic acid) and analyzed at the Center for Metabolomics and Proteomics (CMSP), University of Minnesota. Peptides were separated on an UltiMate 3000 RSLCnano LC system and analyzed on an Orbitrap Eclipse mass spectrometer (MS) (Thermo Fisher Scientific) in a bottom‐up, data dependent scan mode. We performed gradient separation on a self‐packed C18 column (Dr. Maisch GmbH ReproSil‐PUR 1.9 μm 120 Å C18aq, 100 μm ID × 45 cm length) at 55°C with the following profile: 5% B solvent from 0 to 2 min, 8% B at 2.5 min, 21% B at 40 min, 35% B at 75 min and 90% B at 77 min with a flowrate of 400 nL/min from 0 to 2 min and 315 nL/min from 2.5 to 77 min, where solvent A was 0.1% formic acid in water and solvent B was 0.1% formic acid in ACN. We employed the following MS parameters: ESI voltage +2.2 kV, ion transfer tube 275°C; no internal calibration; Orbitrap MS1 scan 120 k resolution in profile mode from 380 to 1400 *m/z* with 50 msec injection time; 100% (4 × 10E^5^) automatic gain control (AGC); MS2 was triggered during a 3 s cycle on precursors with 2–5 charges above 5000 counts. MS2 settings: high energy collisional dissociation (HCD); 1.2 Da quadrupole isolation window, 30% normalized collision energy, MIPS (monoisotopic peak determination) was set to Peptide; ion trap detection, 35 msec max injection time, 100% (1 × 10E^4^) AGC and 30 s dynamic exclusion duration with ±10 ppm mass tolerance.

### 2.5 Proteomic Data Analysis

Proteomic data were analyzed using MaxQuant (version 2.2.0.0) with the *Homo sapiens* UniProt reference proteome database (UP000005640.fasta) [21, 22]. The database was downloaded from UniProt on May 22, 2022, with 79,071 sequences (20,361 sequences from reviewed Swissprot database and 58,710 sequences from unreviewed Tremble database). Methionine oxidation and protein N‐terminal acetylation were specified as variable modifications, while cysteine carbamidomethylation was set as a fixed modification. The maximum number of modifications per peptide was limited to two. Enzyme specificity was set to Trypsin/P with a maximum of one missed cleavage allowed. The false discovery rate (FDR) for proteins, peptides and modifications was all set at 1% with the minimum score of 40 for modified peptides. Minimum numbers of unique and razor peptides were set at 0 and 1 by default. Label‐free quantification (LFQ) was enabled, and the match‐between‐runs feature was applied for identification [23]. LFQ values for each sample were normalized using the DESeq2 R package (v1.44.0). Prior to normalization, protein intensity values were scaled by a factor of 1 × 10^6^ to reduce the influence of extremely high‐abundance proteins. Scaled values were then rounded to integers, as required by DESeq2. Mass spectrometry often has a narrower dynamic range for quantification compared to digital counting of RNA seq. Also 40%–50% increase in rate limiting enzyme can shift a signaling cascade. Due to post transcriptional regulation, higher fold change in mRNA level might only result in much smaller change in protein level. Log2 fold‐change threshold of 0.5 was used to detect differentially abundant proteins. Age covariate was corrected in the design command line. Variance‐stabilizing transformation (VST), normalized counts were subsequently used for downstream machine‐learning analyzes. Proteomic data from pediatric and adult cohorts generated across different laboratories were analyzed using the limma R package (v3.60.6) was included as a covariate in the linear model to account for technical and platform‐specific variability, and empirical Bayes variance moderation was applied to improve robustness. This approach minimizes assay‐related bias and enables reliable cross‐cohort comparisons.

### 2.6 Machine Learning

A two‐stage machine‐learning framework was implemented to decouple feature ranking from multivariate model optimization. In the first stage, variable importance scores derived from the learning vector quantization (LVQ) algorithm (R packages: caret v6.0.94 and BCLench v2.1.3) were used to quantify the marginal contribution of each protein to class separation in a supervised setting. LVQ estimates feature relevance based on prototype–feature distance metrics, allowing stable ranking of proteins in high‐dimensional, low‐sample‐size proteomics data. This step provides a univariate‐informed but model‐aware prioritization of candidate proteins.

In the second stage, feature selection was performed using cross‐validated model training (R packages: caret v6.0.94 and BCLench v2.1.3) to identify a minimal subset of proteins whose joint representation maximizes classification performance. This multivariate optimization accounts for feature interactions and redundancy, enabling selection of complementary protein combinations that outperform individually ranked features. By separating importance estimation from subset selection, this approach reduces overfitting, improves generalizability, and yields interpretable yet high‐performing biomarker panels. Functional analysis including biological pathways and processes was curated with the implementation of different databases such as Reactome and Biological process (Gene Ontology).

### 2.7 V‐Plex Based Inflammatory Protein Level Measurement

Levels of serum proteins CRP (C‐Reactive Protein), FGF (Fibroblast Growth Factor), ICAM‐1 (InterCellular Adhesion Molecule‐1), IFN (InterFeroN)‐γ, IL (InterLeukin)‐1β, IL‐1RA, IL‐6, IL‐8, MMP (Matrix MetalloProteinase)‐1, MMP‐2, MMP‐7. MMP‐9, Pentraxin 3, pro‐MMP9, RAGE (Receptor for advanced Glycation Endoproducts), S100A12, SAA (Serum Amyloid A), TNF (Tumor Necrosis Factor)‐α, TNF‐RI, TNF‐RII, VEGF (Vascular Endothelial Growth Factor)‐A, and VEGF‐D were measured by using customized V‐plex platform‐based ELISA (Meso Scale Discovery; Gaithersburg, Maryland) per the company protocol.

### 2.8 Statistical Analysis

The data are shown as means ± SDs. A one‐way ANOVA with Bonferroni's post hoc analysis and Student's unpaired *t*‐test were used to assess statistical significance with *p* value < 0.05.

## 3 Results

### 3.1 Proteomic Profiling of Plasma From Pediatric Sepsis Patients in Acute and Recovery Phases

**FIGURE 1:**
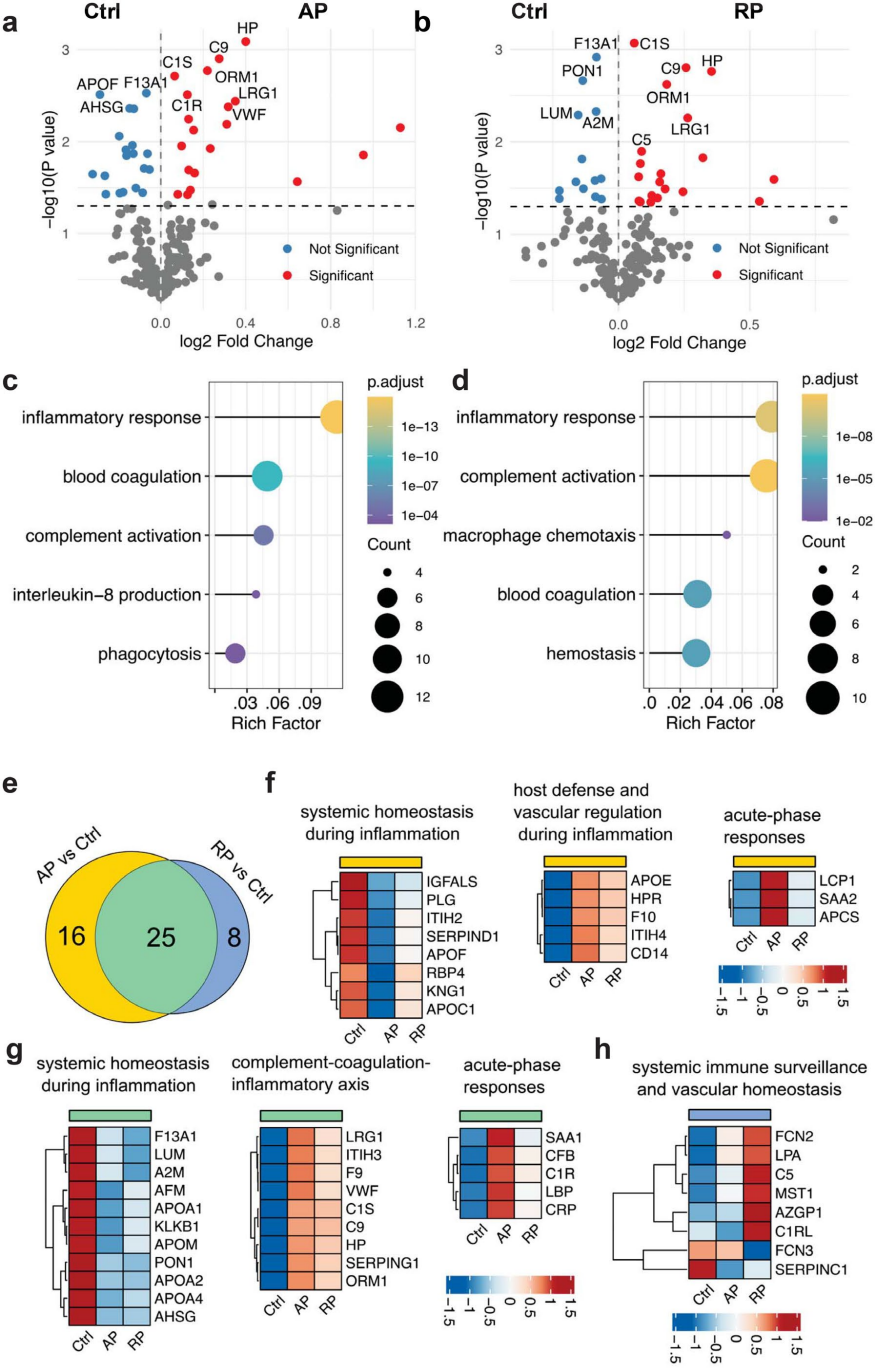
Differential plasma proteomic profiles in pediatric sepsis patients. (a) Volcano plot showing differentially abundant (DA) plasma proteins in the acute phase (AP) of pediatric sepsis compared to controls (Ctrl). Red dots indicate significantly upregulated or downregulated proteins (*p* < 0.05); blue dots indicate non‐significant proteins (*p* > 0.05). (b) Volcano plot of DA proteins in the recovery phase (RP) compared to Ctrl. (c, d) Gene Ontology (GO) enrichment analysis of biological processes for DA proteins in AP (c) and RP (d) relative to Ctrl. Inflammatory response and complement activation pathways are prominently upregulated in both phases. (e) Venn diagram displaying the number of overlapping and unique DA proteins between AP vs. Ctrl and RP vs. Ctrl comparisons. Unique proteins were defined as proteins that were significantly present in AP vs. Ctrl or RP vs. Ctrl alone. (f) Heatmap of proteins uniquely upregulated or downregulated in the AP versus Ctrl comparison. (g) Heatmap of overlapping DA proteins shared between AP and RP relative to Ctrl. (h) Heatmap of proteins uniquely upregulated or downregulated in the RP versus Ctrl comparison.

To investigate systemic responses in pediatric sepsis, we analyzed plasma proteomic profiles from six pediatric sepsis patients at both the acute phase (AP) and recovery phase (RP), alongside five age‐matched controls (Ctrl). Their demographic information was noted in Table 1. Neutrophil counts were significantly elevated in the sepsis group (median 7.44 × 10^3^ vs. 2.75 × 10^3^ cells/μL, *p* = 0.009). Platelet count was significantly lower in the sepsis group (median 92.5 × 10^3^ vs. 327 × 10^3^ cells/μL, *p* = 0.004). Mass spectrometry–based proteomics identified a total of 316 proteins (Table S1), among which were 41 differentially abundant (DA) proteins in AP compared to Ctrl, including 22 upregulated and 19 downregulated proteins (Figure 1a). In the RP vs. Ctrl comparison, 33 DA proteins were found, with 20 upregulated and 13 downregulated (Figure 1b).

**TABLE 1:** Characteristics of enrolled patients.

Subject	Age (year)	Gender	Diagnosis	pSOSF‐AP	pSOFA‐RP	WBC (×10^3^/uL)	Neutrophil (×10^3^/uL)	Monocyte (×10^3^/uL)	Eosinophil (×10^3^/uL)	Basophil (×10^3^/uL)	Lymphocyte (×10^3^/uL)	Platelet (×10^3^/uL)	Hemoglobin (g/dL)
SP1	1	Female	Bronchiolitis	6	2	15.17	12.62	1.05	0.01	0.01	1.09	88	8.7
SP2	15	Female	Toxic shock syndrome	12	6	10.74	5.18	3.3	1.98	0	0.1	30	11.2
SP3	3	Female	Aspiration pneumonia	7	3	21.94	20.78	0	0	0	0.77	36	10.5
SP4	0.7	Male	RSV infection	3	2	6.09	3.45	0.72	0.02	0.02	1.8	102	12.1
SP5	15	Male	Otomastoiditis	3	2	14.27	9.21	1.08	0.09	0.04	3.56	125	8
SP6	15	Male	Candidiasis	8	4	6.48	5.66	0.27	0	0.03	0.43	97	14.9
HC1	0.3	Male		0	0	8.57	1.29	0.38	0.08	0.15	6.68	392	10.7
HC2	10	Female		0	0	6.19	2.85	0.51	0.19	0.04	2.58	327	14
HC3	6	Male		0	0	6.85	2.75	0.66	0.04	0.66	3.23	305	11.9
HC4	0.8	Female		0	0	9.55	2.24	0.5	0.41	0	6.31	500	11.5
HC5	9	Female		0	0	7.49	4.92	0.7	0.2	0.03	1.62	325	13.5

Abbreviations: AP, acute phase; HC, healthy control; RP, recovery phase; RSV, respiratory syncytial virus; SP, sepsis; WBC, white blood cells.

Gene Ontology enrichment analysis of biological processes revealed significant enrichment in pathways related to the inflammatory response and complement activation in both AP and RP groups (Figure 1c,d), which may imply that inflammatory and pathogen‐clearing processes remain active even during recovery.

To explore the overlap in protein expression patterns, we generated a Venn diagram, which revealed 25 shared DA proteins between AP vs. Ctrl and RP vs. Ctrl comparisons (Figure 1e). In Venn diagram, we referred proteins that were statistically significant in one comparison alone (16 proteins in AP vs. Ctrl, or 8 proteins in RP vs. Ctrl) as unique proteins. Proteins that were uniquely or commonly upregulated or downregulated across the two previous comparisons are listed in Table S2. Heatmaps of overlapping and unique proteins of similar functions (Figure 1f–h) highlighted distinct proteomic signatures. Notably, several proteins associated with systemic inflammation were prominently upregulated in AP compared to both RP and Ctrl (Figure 1f,g). These included acute‐phase proteins such as SAA1 (serum amyloid A1), SAA2, CRP (C reactive protein), APCS (amyloid P component, serum), LBP (lipopolysaccharide binding protein), CFB (complement factor B), C1R (complement component 1r), VWF (von Willebrand factor), F9 (coagulation factor 9), and F10 (coagulation factor 10), which may infer ongoing immune activation and complement‐coagulation interplay during acute illness. In contrast, proteins involved in immune surveillance and vascular homeostasis, including AZGP1 (alpha‐2 glycoprotein 1, zinc‐binding), C1RL (complement C1r‐like protein), and MST1 (macrophage stimulating 1), were upregulated in RP compared to AP and Ctrl (Figure 1h), suggesting partial restoration of immune equilibrium during recovery. Overall, these findings underscore the persistence of inflammatory and complement activity throughout the sepsis course, with partial resolution during the recovery phase.

To characterize phase‐specific signatures, we performed a direct comparison between AP and RP, which identified 17 differentially expressed proteins (Figure S1a). Differentially abundant proteins in AP included SAA1, C1QA, C1R, APCS, ACTB (actin beta), and SERPINF2 (Serpin family F member 2). In contrast, differentially abundant proteins in RP included C5, APOH, APOC2, C1RL, APOC4.

### 3.2 Dynamic Protein Profile Changes in Acute and Recovery Phases of Pediatric Sepsis: Machine Learning–Based Biomarker Discovery

**FIGURE 2:**
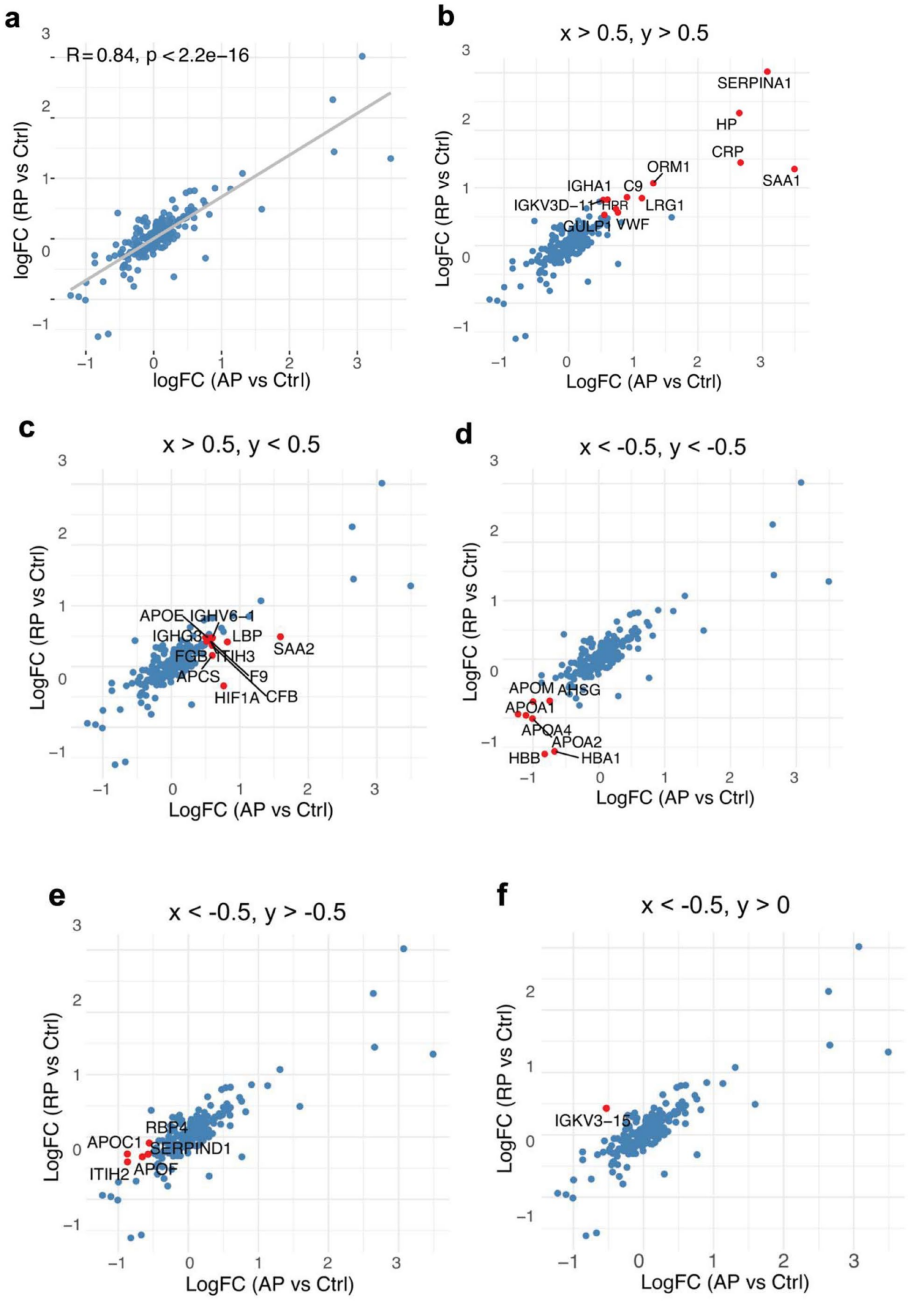
Dynamic changes in plasma protein expression across acute and recovery phases of pediatric sepsis. (a) Linear regression analysis of log_2_ fold changes in protein expression between acute phase (AP) and recovery phase (RP) samples relative to Ctrl. (b–f) Scatter plots showing proteins significantly upregulated (log_2_FC > 0.5) in both AP and RP compared to controls (Ctrl) as indicated on the top of each subfigure, *x* and *y* represent logFC(AP vs. Ctrl) and logFC(RP vs. Ctrl), respectively.

To assess the global trend of protein expression changes across sepsis phases, we first performed linear regression analysis using all upregulated proteins (Figure 2a). This analysis revealed a strong positive correlation (*R* = 0.84) between protein expression in AP and RP, supporting the notion of a sustained inflammatory signature throughout the disease course. This persistent response may reflect a protective mechanism against secondary infections.

We next focused on proteins that were highly upregulated (log_2_fold change > 0.5) in both AP and RP relative to controls (Figure 2b). Among these, key proteins included SERPINA1 (serine protease inhibitor A1, alpha‐1 antitrypsin), HP (haptoglobin), CRP, SAA1, ORM1 (orosomucoid 1), C9, LRG1 (leucine‐rich alpha‐2 glycoprotein 1), VWF, HPR (haptoglobin‐related protein), GULP1 (PTB domain‐containing engulf adaptor protein 1), and IGKV3D‐11 (immunoglobulin kappa variable 3D‐11). Notably, SERPINA1 was markedly elevated in both phases. As It targets neutrophil elastase in vivo to inhibit neutrophil extracellular traps (NETs) formation [24], the data might highlight its potential role in regulating inflammation and limiting tissue damage during sepsis. In contrast, several acute‐phase proteins including SAA2, F9, CFB, HIF1A (hypoxia‐inducible factor 1‐apha) showed high expression during AP (log_2_FC > 0.5) but returned to baseline or lower levels in RP (log_2_FC < 0.5), as shown in Figure 2c.

Conversely, proteins consistently downregulated in both AP and RP (log_2_FC < 0) were primarily apolipoproteins (Figure 2d–f), suggesting disruptions in lipid transport and systemic homeostasis during sepsis.

**FIGURE 3:**
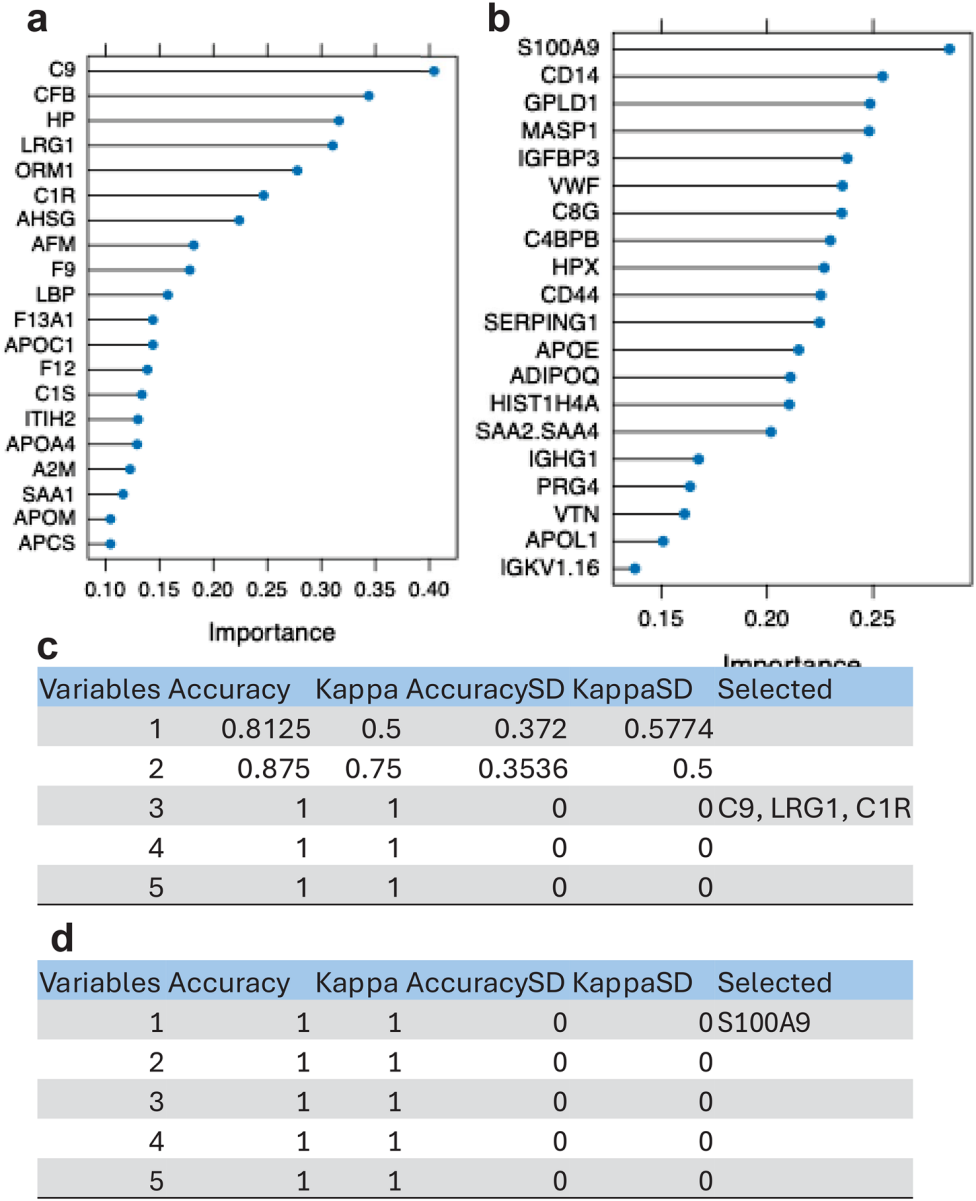
Machine learning–based selection of plasma biomarkers in acute and recovery phases. (a, b) Random Forest variable importance analysis (mean decrease in accuracy) identifying proteins most predictive of the acute phase (AP; a) and recovery phase (RP; b) relative to controls. (c, d) Cross‐validation–based feature selection identifying the optimal combination of proteins for classifying AP (c) and RP (d) relative to controls.

To identify potential biomarkers capable of distinguishing between AP and RP, we applied supervised machine learning (ML) techniques. Random forest–based variable importance analysis, quantified using the mean decrease in classification accuracy, was first used to rank proteins according to their contribution to discriminating AP from control samples (Figure 3a). This analysis identified C9, CFB, HP, LRG1, ORM1, and C1R as top predictive features.

To further refine biomarker selection and account for multivariate feature interactions, we subsequently performed cross‐validation–based feature selection, which identified C9, LRG1, and C1R as the most informative protein combination for AP classification (Figure 3b).

An analogous ML workflow was applied to the RP group (Figure 3c,d), where random forest variable importance analysis ranked S100A9 as the most predictive candidate biomarker distinguishing the recovery phase from controls.

We further applied the two machine learning approaches to the direct AP versus RP comparison and identified C1RL, APOH, and ACTB as the top candidate proteins distinguishing AP from RP (Figure S1b,c). The discriminatory performance of this three‐protein panel was validated using ROC curve analysis, achieving an AUC of 0.92 (Figure S1d).

### 3.3 Cytokine and Chemokine Profiling in Acute and Recovery Phases of Pediatric Sepsis

**FIGURE 4:**
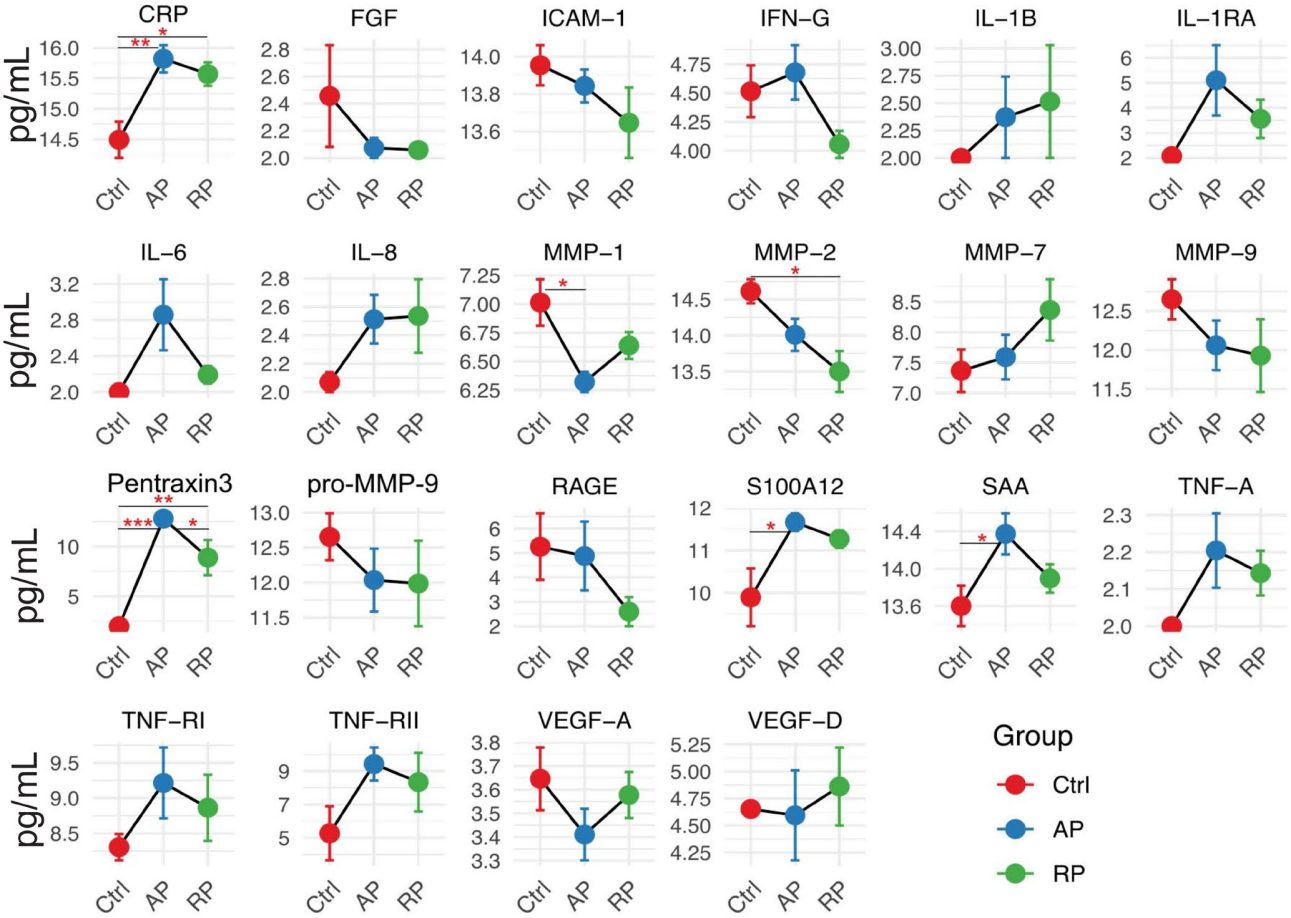
Inflammatory mediators' profiles in pediatric sepsis during acute and recovery phases. Line pots depicting plasma levels of selected cytokines, chemokines, and vascular/tissue remodeling proteins were measured in controls (Ctrl, red, *n* = 5), acute sepsis phase (AP, blue, *n* = 6), and recovery phase (RP, green, *n* = 6). Data are presented as mean ± SD. Statistical significance was determined between groups (*p < 0.05, **p < 0.01).

We profiled a panel of inflammatory mediators in plasma from the three study groups Ctrl, AP, and RP (Figure 4). Acute phase proteins, including CRP, SAA, and S100A12, were significantly elevated in AP and remained partially elevated in RP, which may suggest sustained systemic inflammation, consistent with the result from the proteomics. Pro‐inflammatory cytokines such as IL‐6, TNF‐α, IL‐1β, and IFN‐γ showed a marked upregulation trend in AP, while IL‐1RA, an anti‐inflammatory cytokine, also increased, which may indicate a counter‐regulatory response. Consistent elevation of IL‐8 in AP and RP confirmed the proteomics pathways seen in Figure 1c,d.

Interestingly, endothelial and vascular markers such as ICAM‐1 (intercellular adhesion molecule 1), VEGF‐A (vascular endothelial growth factor‐A), VEGF‐D, and FGF (fibroblast growth factor) numerically showed reduced levels in both AP and RP compared to controls (Figure 4). ICAM‐1 data is consistent with another pediatric sepsis study [25]. While our proteomics indicated vascular activation (Figure 1f), this result might suggest that vascular repair or angiogenesis was impaired. Similarly, decreased matrix metalloproteinases (MMP‐1, −2, and −9) (Figure 4), which may suggest suppression of extracellular matrix degradation and tissue remodeling, is in line with the data of VEFG‐D and FGF. TNF receptors (TNF‐RI and TNF‐RII) were also upregulated in AP and RP, which may suggest ongoing modulation of TNF‐mediated signaling. Pentraxin 3 was significantly upregulated in AP and RP (AP > RP). Pentraxin 3 activates the complement pathway [26], in line with the complication activation seen in sepsis (Figure 1g).

Together, these profiles complement the proteomic data and emphasize the persistent immune activation and vascular involvement throughout sepsis progression and partial resolution.

### 3.4 Comparison of Protein Signatures Adult and Pediatric Sepsis

**FIGURE 5:**
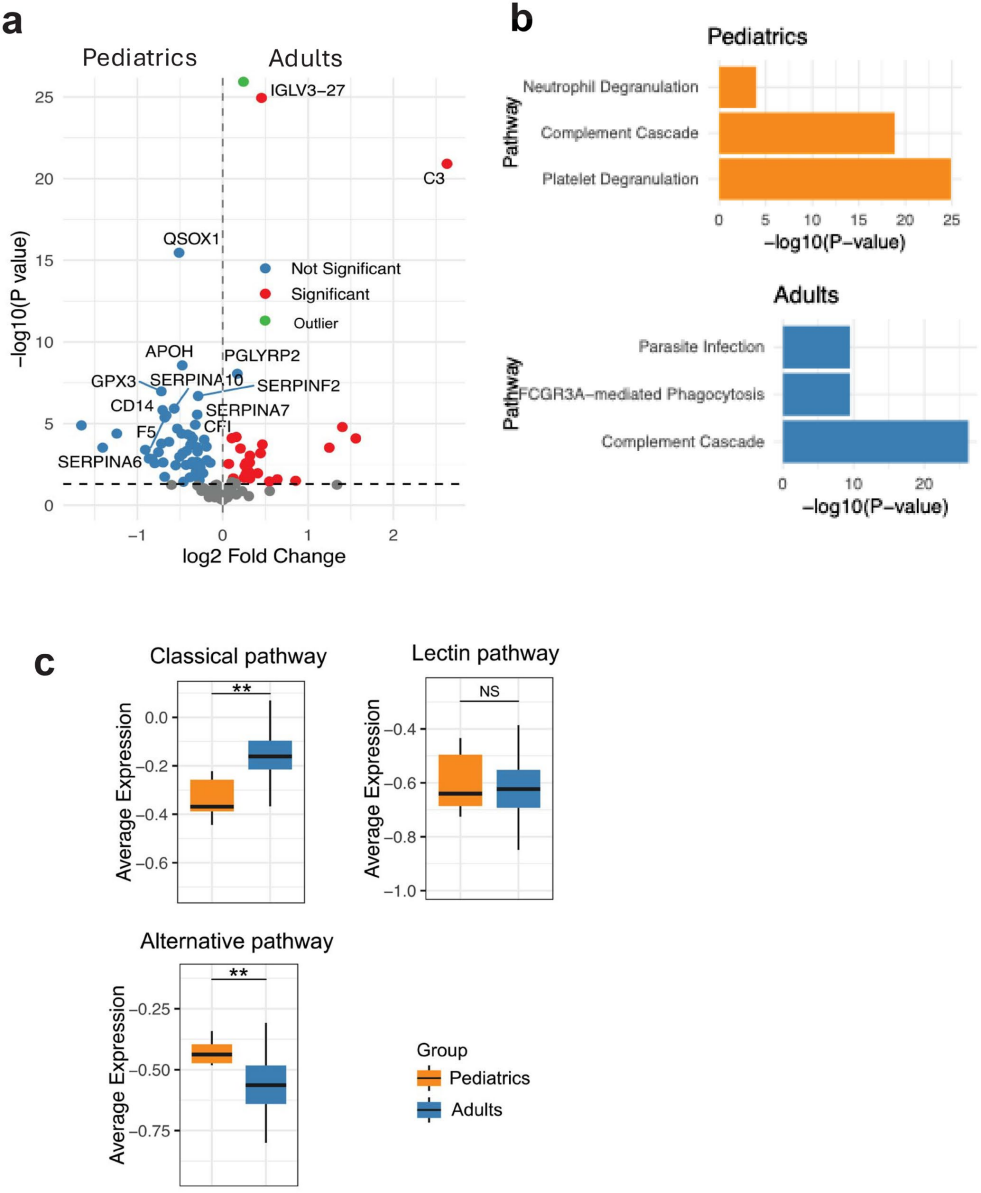
Comparative proteomic profiling between adult and pediatric sepsis. (a) Volcano plot of DA proteins in the adult compared to pediatric sepsis. Green dot indicates proteins with –log10(*p* value) > 26, which are displayed separately to enhance volcano plot visualization. These proteins include HBA2, FN1, IGHV3‐15, IGLV7‐43. ALB, SERPINA3 and RBP4. (b) Bar plots of Reactome enrichment analysis of biological pathways for DA proteins in adult relative to pediatric sepsis. (b) Box plots depicting the levels of three complement pathways in adult relative to pediatric sepsis. Data are presented as mean ± SD. Statistical significance was determined between groups (**p < 0.01).

To further investigate age‐related differences in sepsis, we analyzed publicly available proteomic data from adult sepsis subjects (*n* = 204) (Accession number: PXD055932) [27] and compared them with pediatric sepsis profiles. Differential expression analysis identified 121 DA proteins between adult and pediatric sepsis (Figure 5a), including 47 proteins overexpressed in adults and 74 proteins overexpressed in pediatric patients. Subsequent biological pathway enrichment analysis highlighted the top three pathways for each condition (Figure 5b). Notably, the complement cascade emerged as a shared pathway between both groups. Given its central role in immune defense and inflammation, we performed a focused comparison of the three complement activation pathways across adult and pediatric sepsis (Figure 5c). The classical pathway was significantly upregulated in adults compared to pediatric patients, whereas the alternative pathway showed higher activity in pediatric subjects. The lectin pathway did not exhibit a marked difference between the two groups. These findings suggest age‐specific complement activation patterns that may contribute to distinct immune responses in sepsis.

### 3.5 Comparison of Protein Signatures Between Sepsis and Sterile Inflammation

**FIGURE 6:**
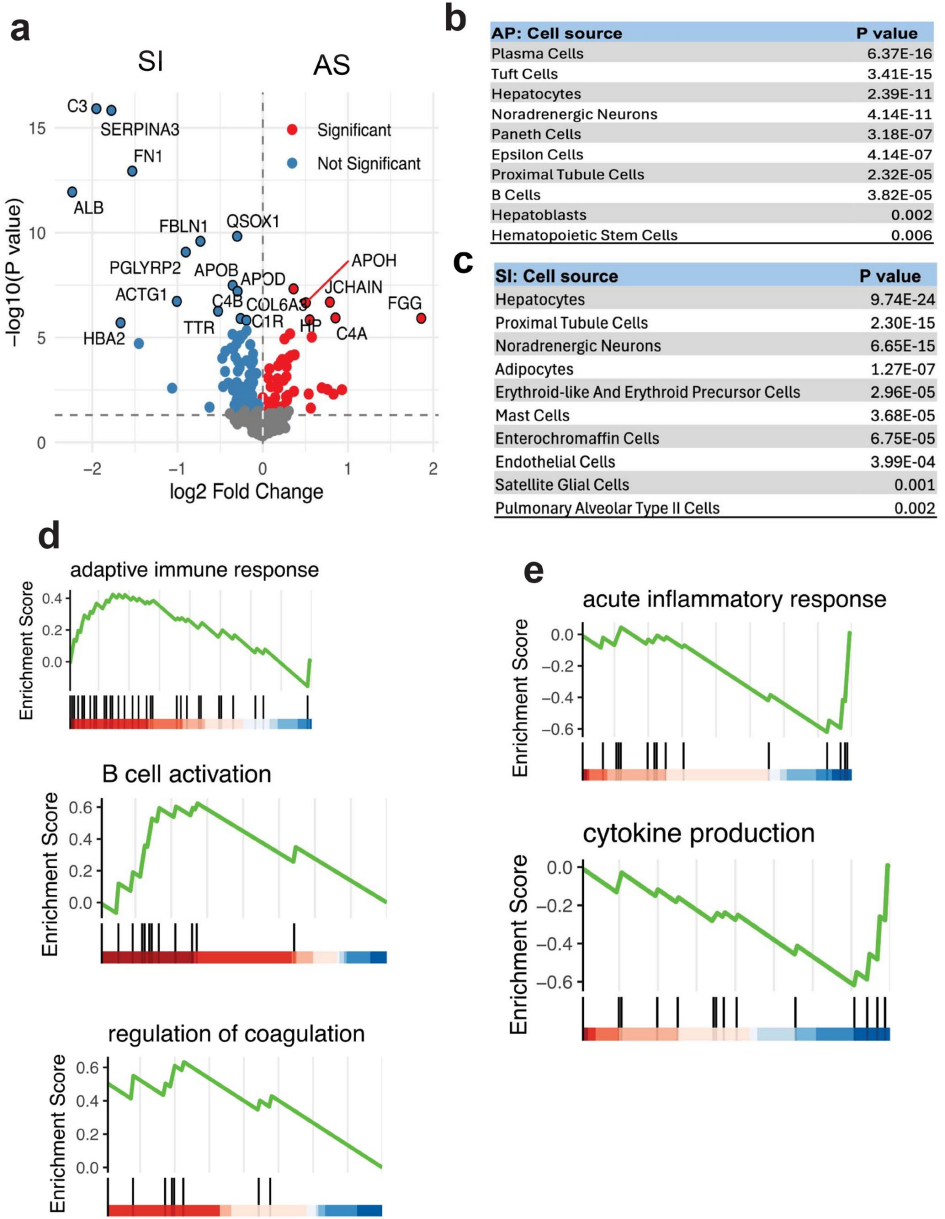
Comparative proteomic profiling between acute sepsis (AS) and sterile inflammation (SI). (a) Volcano plot showing differentially abundant (DA) plasma proteins between AS and SI groups. Red dots represent proteins significantly upregulated in AS (right) and SI (left) (*p* < 0.05); blue dots indicate non‐significant proteins (*p* > 0.05). (b, c) Cell source enrichment analysis of DA proteins from (a), revealing the dominant immune cell types associated with AS (b) and SI (c). (d, e) Gene Set Enrichment Analysis (GSEA) of proteins upregulated in AS relative to SI (d) and upregulated in SI relative to AS (e).

To distinguish sepsis from non‐infectious systemic inflammation, we recruited 10 patients (age 2.4 ± 1.5 years) undergoing congenital heart surgery as representatives of sterile inflammation (SI) and compared their plasma proteomic profiles with those from acute pediatric sepsis (AS) donors. A volcano plot revealed 145 DA proteins between AS and SI (Figure 6a), with 49 proteins upregulated in AS and 96 upregulated in SI.

To explore the cellular origins of these DA proteins, we performed cell source enrichment analysis (Figure 6b). Notably, proteins upregulated in the AS group were predominantly associated with adaptive immune cells, including plasma cells and B cells, while proteins upregulated in SI were linked to innate immune cells, such as mast cells. This suggests a fundamental immunological distinction: sepsis appears to involve a stronger adaptive immune response, whereas sterile inflammation is dominated by innate immunity.

Further gene set enrichment analysis (GSEA) supported this distinction. In AS, pathways such as adaptive immune response, B cell activation, and regulation of coagulation were significantly upregulated (Figure 6c). In contrast, the SI group showed enrichment for pathways related to acute inflammatory response and cytokine production (Figure 6d).

To evaluate the overall expression trends, we conducted a linear regression analysis using all upregulated proteins in AS vs. SI (Figure S2a). This revealed a weak positive correlation (*R* = 0.3), underscoring the divergent proteomic landscapes of infectious and sterile inflammation.

We then focused on proteins that were highly upregulated in both AS and SI (log_2_fold change > 1) compared to controls (Figure S2b). Common inflammatory markers such as SERPINA1, SAA1, SAA2, CRP, C9, LRG1, and VWF were upregulated in both conditions, which may hint that these represent general markers of systemic inflammation rather than disease‐specific biomarkers. As seen in previous comparisons, apolipoproteins were consistently downregulated in both AS and SI (log_2_FC < 0) (Figure S2d,e), further pointing to their role in systemic metabolic shifts during inflammation.

In contrast, a subset of proteins, including HPR, LPA, F9, IGHV6‐1, HP, ORM1, and GULP1, were specifically upregulated in AS (log_2_FC > 1) but not elevated in SI (log_2_FC < 1) (Figure S2c). These proteins are functionally linked to immune regulation and vascular homeostasis during inflammation, highlighting their potential as sepsis‐specific biomarkers.

Conversely, a group of proteins, including ALB (albumin), C3, SERPINA3, HBA2 (hemoglobin, alpha‐2), FN1 (fibronectin 1), ACTG1 (actin gamma 1), PGLYRP2 (peptidoglycan recognition protein 2), and FBLN1 (fibrillin‐1), were uniquely upregulated in SI (Figure S2f), but not in AS. These proteins were associated with innate immune regulation and extracellular matrix remodeling, suggesting a distinct tissue repair–driven response in sterile inflammation. Finally, SERPINA4 was slightly upregulated in SI and downregulated in AS in comparison to Ctrl (Figure S2e).

Additionally, we compared our dataset with a published study by Shubin et al. [28], which performed a similar analysis of SI (*n* = 28) versus AS (*n* = 35) in a pediatric cohort. Although Shubin et al. employed a different proteomics platform (aptamer‐based), we identified 18 shared differentially abundant proteins (Figure S3a). Among these, FGG and HP exhibited consistent overexpression in AS compared to SI across both datasets (Figure S3b); as noted above, these acute‐phase proteins contribute to tissue protection during injury and infection. In contrast, ALB, HBA2, FN1, CAT (catalase), SERPINA4 (Serpin Family A Member 4), PROC (Vitamin K dependent plasma glycoprotein), PRDX6 (peroxiredoxin‐6), and AFM (afamin) showed a consistent under‐expression trend in both datasets. These plasma and extracellular matrix proteins play key roles in host defense under stress, acting at the interface of oxidative stress regulation, vascular protection, coagulation balance, and tissue repair.

## 4 Discussion

Our multi‐phase proteomic analysis reveals a sustained inflammatory and complement activation signature in pediatric sepsis, with partial resolution during recovery. Acute‐phase samples showed robust upregulation of classical acute‐phase proteins (e.g., CRP, SAA1/2, APCS), which may infer systemic immune activation. Notably, several of these proteins remained elevated in the recovery phase, suggesting prolonged immune perturbation even after clinical improvement [29, 30].

Machine learning–based biomarker discovery identified C9, LRG1, and C1R as top candidates for distinguishing acute sepsis compared to control. LRG1 is a pathogenic mediator implicated in diverse diseases and is upregulated in response to IL‐6 stimulation [31, 32]. In septic mouse models, silencing LRG1 attenuated sepsis‐associated encephalopathy (SAE)–related brain injury by inhibiting tumor growth factor (TGF)‐β1/SMAD signaling [12], suggesting that LRG1 may represent a promising therapeutic target for SAE. S100A9 emerged as a potential biomarker of the recovery phase in sepsis. As a component of the S100A8/A9 heterodimer (calprotectin), S100A9 functions as a damage‐associated molecular pattern (DAMP) capable of acting as both a pro‐inflammatory mediator and an immune regulator through TLR4 (Toll‐like receptor 4) and RAGE signaling [33]. Its elevation during recovery may reflect ongoing chemotactic activity and sustained phagocyte recruitment, processes that facilitate cellular debris clearance and promote tissue repair [34]. Persistent upregulation could also indicate prolonged myeloid cell “priming,” with elevated S100A9 levels maintained for weeks after apparent clinical resolution [35]. Another interesting finding is that apolipoprotein levels were reduced in sepsis. Bracht et al. identified four subtypes based on patients' characteristics where the subpopulation associated with severe sepsis had significantly less apolipoproteins [36]. Lipoproteins are lipid cargo. Apolipoproteins are proteins that bind to lipoproteins on their surface and regulate their trafficking. Lipoproteins can also carry lipopolysaccharide (LPS), so that it is possible that reduction in apolipoprotein levels can lead to less sequestration of LPS with more severity. Comparison between AP and RP demonstrated ACTB as an AP marker and C1RL and APOH as markers for RP. Previously, actin level was shown to be associated with severity of sepsis [37], and APOH was reported as a protective factor in sepsis by binding to and scavenging lipopolysaccharide (LPS) [38].

Our cross‐cohort comparison suggests that complement activation in sepsis follows distinct, age‐dependent patterns: adults preferentially engaged the classical pathway, whereas children showed comparatively stronger alternative pathway activity. This divergence likely reflects developmental differences in immune architecture, including lower antibody titers and reliance on pattern‐recognition pathways in children versus antibody‐driven classical pathway activation in adults, a distinction shaped by the maturation status of the adaptive immune system [39, 40, 41]. Bracht et al. identified three adult sepsis subgroups with three different sepsis severity [36]. As sepsis severity increased, most complement components showed reduction, while complement factor D (CFD) levels increased. As primary function of CFD is to cleave factor B once it binds to C3b, they claimed that elevated CFD could facilitate alternative pathway. However, CFB seems significantly consumed in most severe sepsis in the dataset, which was rather in line with less alternative pathway activity in adult. Functionally, alternative pathway dominance in children may provide rapid, antibody‐independent pathogen clearance, while classical pathway activity in adults is amplified by pre‐existing IgG and pentraxin‐mediated activation [42, 43]. In line, the alternative complement pathway activation was reported in infants with infection [44]. Age‐tailored complement modulation may enhance precision therapy in sepsis.

Comparison with sterile inflammation revealed a weak positive correlation, implicating distinct proteomic signatures. Sepsis was characterized by adaptive immune cell–derived proteins and enrichment of B cell–related pathways, whereas sterile inflammation showed predominance of innate immune markers and extracellular matrix remodeling. This observation was further supported by comparison with the dataset from Shubin et al. [28], in which several tissue remodeling proteins, including FN1 and SERPINA4 [45, 46], were consistently shared between the two datasets. It was shown that fibronectin‐based interventions can mitigate sepsis‐associated tissue injury. In a cecal ligation and puncture‐induced murine sepsis model, treatment with recombinant human fibronectin type III homology C fragment (rhFNHC‐36) improved survival, enhanced bacterial clearance, and alleviated histopathological damage in liver, spleen, and lung [47]. These protective effects were linked to reduced systemic inflammation (e.g., lower IL‐6), restored macrophage chemotaxis, and attenuated PD‐L1 upregulation on macrophages. Together, these observations support a role for FN1 not only as a structural extracellular matrix protein but also as a regulator of immune–tissue interactions in sepsis. In line with the role of FN1 in tissue remodeling, SERPINA4 (kallistatin) also emerges as a key regulator of repair and protection during sepsis [48]. SERPINA4 has been shown to suppress HMGB1 (high mobility group box 1)/TLR4‐driven inflammation while preserving endothelial nitric oxide synthase activity, thereby maintaining vascular integrity. Beyond its anti‐inflammatory effects, it limits apoptosis and supports tissue repair, ultimately enhancing bacterial clearance and reducing organ injury. These findings suggest that SERPINA4 not only protects against sepsis‐induced damage but also contributes to the remodeling processes required for recovery. Overall, continuous monitoring of FN1 and SERPINA4 levels could help predict disease severity and tissue damage in septic patients.

Our study has several limitations that should be acknowledged. First, the relatively small sample size, especially in the pediatric cohort, may limit the generalizability of the findings and reduce statistical power. Second, potential confounding factors such as heterogeneity in underlying conditions, treatments, and time points of sample collection may have influenced protein expression patterns. Additionally, the comparison with adult datasets relied on publicly available proteomics generated using different platforms and methodologies, which may introduce technical variability. In addition, cohort characteristics and clinical severity might influence the observed differences. Third, while our machine learning–based biomarker discovery identified candidate proteins for acute and recovery phases, these require validation in larger, independent cohorts and functional studies to confirm their diagnostic and mechanistic relevance. Thus, our data needs to be validated in larger cohorts. Lastly, we did not use albumin depletion in proteomics, which may limit our resolution of proteomics. Collectively, our study is exploratory in nature. Nonetheless, our findings provide a detailed molecular portrait of pediatric sepsis progression and resolution, offering insights into immune dynamics and candidate biomarkers for clinical translation.

## Author Contributions

F.A. performed proteomics data analysis and drafted the manuscript. Y.‐C.S. contributed to proteomics analysis. E.M. coordinated patient consent and sample collection for sepsis and congenital heart disease (CHD) cohorts. H.V.P. facilitated patient consent for the CHD cohort. S.K. supported patient consent and sample acquisition for both sepsis and CHD groups. L.H. conducted proteomics experiments. Y.C. contributed to proteomics experimentation and data generation. K.Y. oversaw sample collection, patient screening, plasma handling, and multiplex immunoassays; also contributed to manuscript editing and secured funding.

## Funding

This study was supported by R01GM148392 (K.Y.) R21HD109119 (K.Y.), CHMC Anesthesia Foundation (K.Y.) The Orbitrap Eclipse instrumentation platform used in this work was purchased through High‐end Instrumentation Grant S10OD028717 from the NIH.

## Ethics Statement

This study was conducted in accordance with the ethical standards of the Institutional Review Board at Boston Children's Hospital, which approved all protocols involving human participants.

## Consent

Written informed consent was obtained from a parent or legal guardian for each participant prior to enrollment. All procedures were approved by the Institutional Review Board at Boston Children's Hospital.

## Conflicts of Interest

The authors declare no conflicts of interest.

## Supporting information


**Data S1:** fba270098‐sup‐0001‐FigureS1‐S3.pdf.

**Table S1:** Annotated proteins from proteomics.

**Table S2:** Uniquely and shared upregulated/downregulated proteins in AP vs. Ctrl and RP vs. Ctrl comparisons.

## Data Availability

Raw data supporting the findings of this study will be shared upon reasonable request.
