# Supplementary material for: Plasma Proteomic Signatures of Pediatric Sepsis Reveal Persistent Inflammation and Phase‐Specific Biomarkers
Source: FASEB Bioadv. 2026 Mar 9;8(3):e70098. doi: 10.1096/fba.2026-00006 (PMC12972194; doi:10.1096/fba.2026-00006)
Supplement: Supplementary file 1 — Data S1: fba270098‐sup‐0001‐FigureS1‐S3.pdf. [file FBA2-8-e70098-s001.pdf]

a

Protein	log2(FC)
SAA1	0.59
C1QA	0.34
HBD	0.23
IGHV1-46	0.13
APCS	0.11
ACTB	0.07
C1R	0.05
SERPINF2	0.03
KNG1	-0.03
C5	-0.05
APOH	-0.09
APOC2	-0.12
IGKV1-8	-0.19
RBP4	-0.22
C1RL	-0.22
APOC4	-0.23
IGKV3-15	-0.39

b

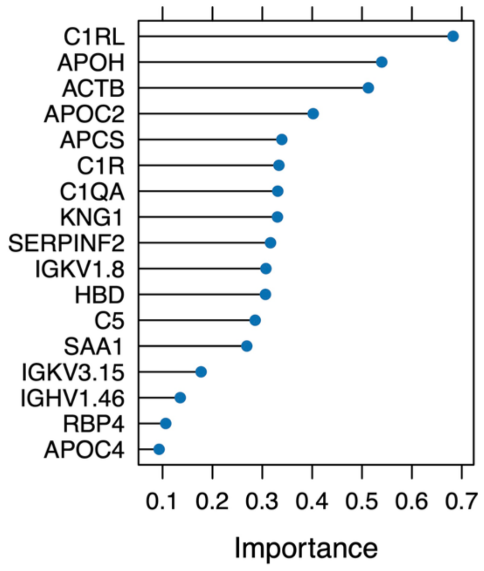

c

Variables	Accuracy	Kappa	AccuracySD	KappaSD	Selected
1	0.625	0.3333	0.4432	0.5164	
2	0.625	0.3333	0.4432	0.5164	
3	0.8125	0.6	0.372	0.5477	C1RL, APOH, ACTB
4	0.625	0.3333	0.4432	0.5164	
5	0.625	0.3333	0.4432	0.5164	

d

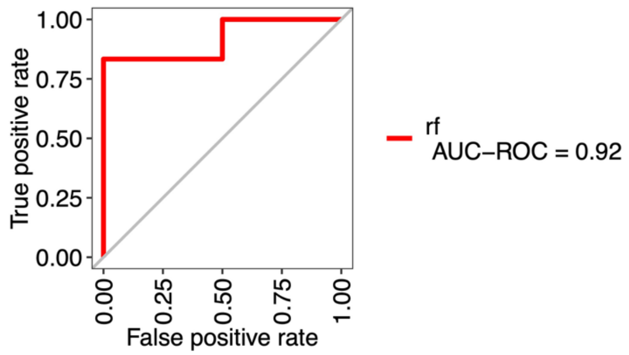

Supplementary Figure 1. Machine learning–based selection of plasma biomarkers distinguishing acute and recovery phases.

(a) Differentially expressed proteins identified in the AP versus RP comparison, shown with corresponding $\log_2$ fold change ($\log_2$FC) values.

(b) Random Forest variable importance analysis (mean decrease in accuracy) highlighting proteins most predictive of the acute (AP) and recovery (RP) phases.

(c) Cross-validation–based recursive feature selection identifying the optimal protein combination for discriminating between AP and RP.

(d) Receiver operating characteristic (ROC) curve analysis demonstrating the classification performance of the three-marker panel (C1RL, APOH, and ACTB), with an AUC of 0.92.

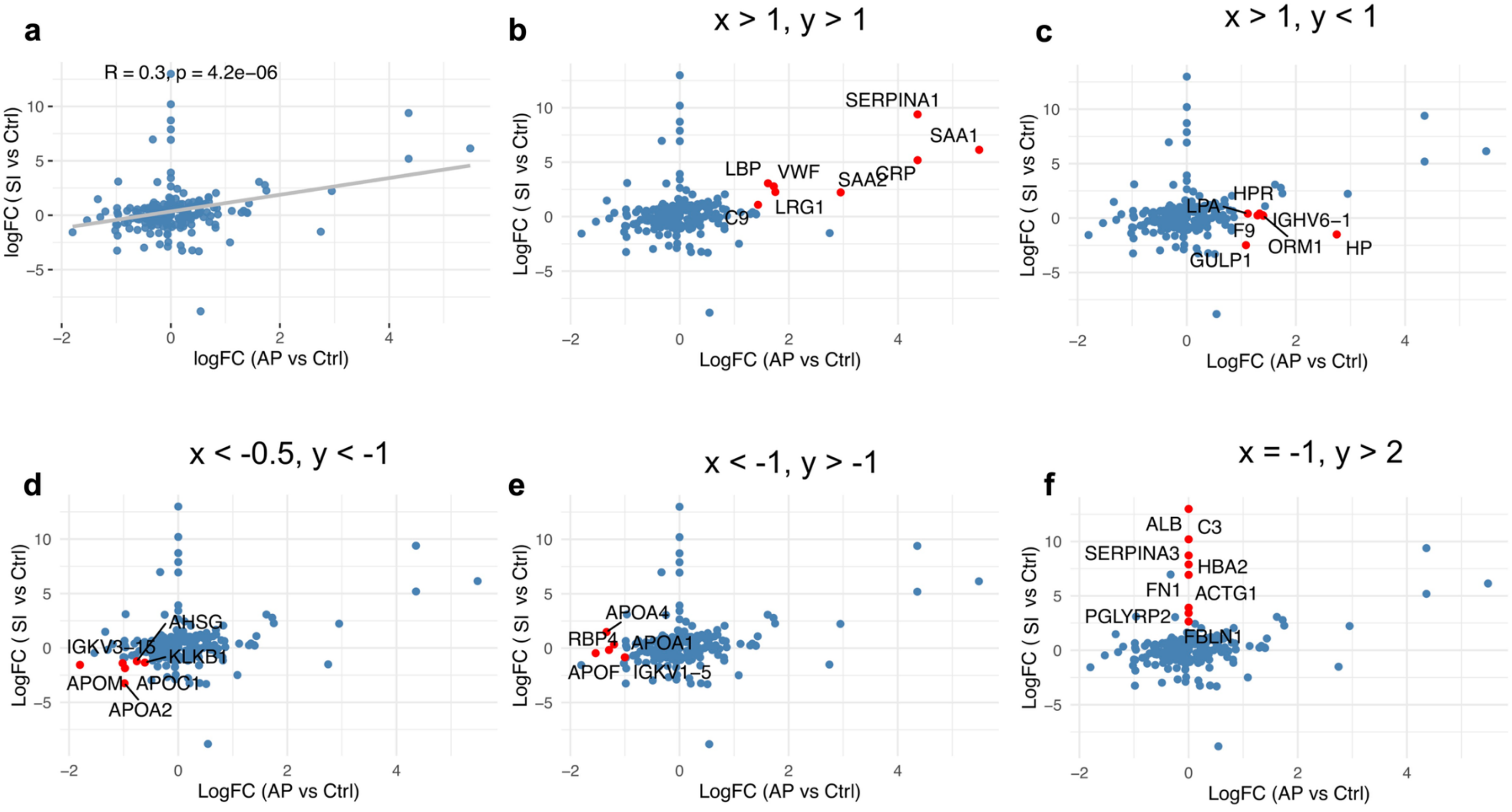

Supplementary Figure 2. Differential expression trends and biomarker prioritization in AS vs. SI

(a) Linear regression of fold-change values (AS vs. Ctrl and SI vs. Ctrl) for all upregulated proteins.

(b–f) Scatter plots showing proteins significantly upregulated ($log_2FC > 0.5$) in both AS and SI compared to controls (Ctrl) as indicated on the top of each subfigure, x and y represent logFC(AS vs Ctrl) and logFC(SI vs Ctrl), respectively.

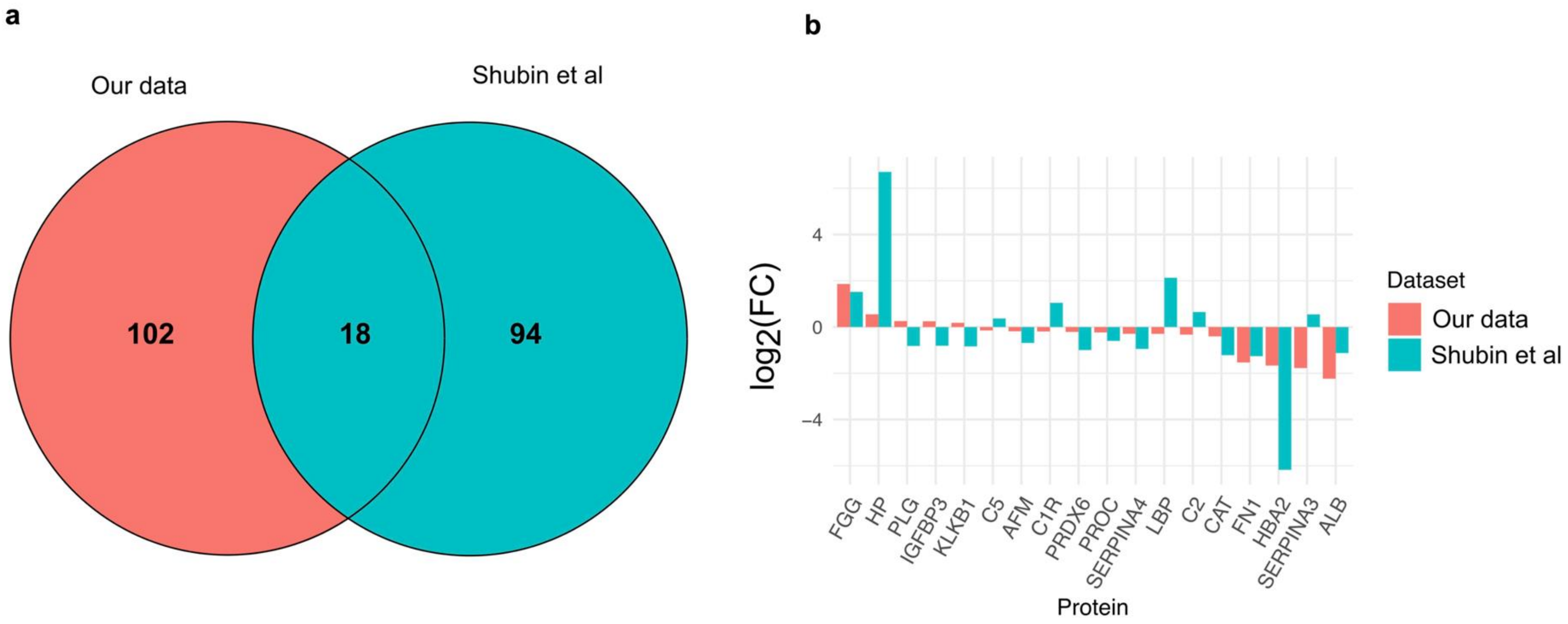

Supplementary Figure 3. AS vs. SI comparison in Shubin et al dataset
(a) Venn diagram depicting the unique and shared differentially abundant proteins between Shubin et al and our data.
(b) Bar plots illustrating the expression patterns of the 18 differentially abundant proteins shared between Shubin et al. and our dataset.
