# Supplementary material for: Plasma Proteomic Signatures of Pediatric Sepsis Reveal Persistent Inflammation and Phase‐Specific Biomarkers
Source: FASEB Bioadv. 2026 Mar 9;8(3):e70098. doi: 10.1096/fba.2026-00006 (PMC12972194; doi:10.1096/fba.2026-00006)
Supplement: Supplementary file 2 — Table S1: Annotated proteins from proteomics. [file FBA2-8-e70098-s003.docx]

**Suppl. Table 1. Annotated proteins from proteomics**

| SERPINA1 | MASP1 | HPX | TGFBI |
| --- | --- | --- | --- |
| IGLV2-18 | RTN4 | F11 | ADIPOQ |
| IGLV3-12 | BCHE | C4BPA | ECM1 |
| IGLV3-10 | TFRC | VTN | HSP90AB4P |
| IGHV4-4 | HBA2 | CAT | ANP32E |
| IGKV2-24 | SERPINA10 | APOB | VASN |
| IGKV1-8 | SERPINA3 | HRG | PI16 |
| IGKV2D-30 | MST1 | IGLV7-43 | CCNB3 |
| IGHV3OR16-12 | CD44 | A1BG | PRG4 |
| IGHV3OR15-7 | STXBP5 | VWF | PGLYRP2 |
| CPB2 | FN1 | GAPDH | COLEC11 |
| TTR | FCGR3A | IGKV1-16 | C14orf142 |
| FCGBP | KLKB1 | SERPINA5 | C1RL |
| ALDOB | SHBG | F13B | GULP1 |
| TNXB | ACTG1 | ICAM1 | PCYOX1 |
| GPX3 | ALDOA | SERPINA7 | SLC27A5 |
| C1S | CNDP1 | SERPIND1 |  |
| SAA2-SAA4 | LCAT | IGKV4-1 |  |
| F5 | PARK7 | C2 |  |
| IGKV3D-11 | APOC2 | S100A9 |  |
| IGHG1 | CFD | APOA4 |  |
| IGHD | APOC1 | CKM |  |
| IGHV3-49 | SBSN | C8A |  |
| PVR | C3 | C8G |  |
| C1QB | PGLYRP2 | BPGM |  |
| DDX39B | ZNF675 | HSP90AA1 | |
| IGHV6-1 | MASP2 | SERPINA6 |  |
| IGHV3-15 | QSOX1 | HSP90AB1 | |
| IGHV3-21 | APOL1 | LPA |  |
| IGHV3-74 | CD5L | CD14 |  |
| IGHV3-43 | N4BP1 | CFH |  |
| IGLL5 | FCN3 | SERPINF2 |  |
| IGKV1D-13 | ATRN | DBH |  |
| IGHV1-69-2 | APOM | C4A |  |
| CRTAC1 | SOD1 | C4B |  |
| GP1BA | CP | SAA1 |  |
| IGHV1-3 | F13A1 | SAA2 |  |
| IGHV1-18 | PNP | IGLC6 |  |
| IGHV3-38 | F2 | IGHV3-30-3 | |
| IGHV5-51 | HP | IGHV4-28 |  |
| IGHV2-70 | HPR | C7 |  |
| IGHA2 | F9 | CLU |  |
| C4A | F10 | MBL2 |  |
| IGLV5-45 | PLG | CETP |  |
| IGHV3-64D | F12 | COL6A3 |  |
| IGHV5-10-1 | CA1 | C6 |  |
| PROS1 | CA2 | LCP1 |  |
| SEPP1 | SERPINC1 | SELL |  |
| IGHG3 | SERPINA1 | NID1 |  |
| IGHA1 | SERPINA3 | CPN1 |  |
| IGHG2 | A2M | B4GALT1 |  |
| IGHG4 | C3 | IGFBP2 |  |
| BCAR1 | C5 | LBP |  |
| ENO1 | TIMP1 | VCAM1 |  |
| HBG1 | CST3 | ORM2 |  |
| C1R | KNG1 | ITIH2 |  |
| IGFBP6 | JCHAIN | ITIH1 |  |
| PDIA4 | IGKV1-33 | PZP |  |
| IGHV3-72 | IGKV1D-39 | C4BPB |  |
| LDHB | IGKV1-17 | CPN2 |  |
| SNCA | IGKV1-5 | IGHV1-2 |  |
| FAT4 | IGKV3-20 | AZGP1 |  |
| SERPING1 | IGKV3-15 | PON1 |  |
| NPM1 | IGLV1-47 | SERPINA4 |  |
| BTF3 | IGLV1-51 | PRDX6 |  |
| AGT | IGLV3-19 | BLVRB |  |
| HSPA5 | IGLV3-1 | PRDX2 |  |
| PKM | IGLV3-25 | CDH5 |  |
| IGFBP3 | IGLV3-27 | IGFALS |  |
| ZSWIM8 | IGLV6-57 | SERPINF1 |  |
| ALDOC | IGHV1-69 | PTGDS |  |
| HIF1A | IGHV1-46 | BTD |  |
| MB | IGHV3-23 | AFM |  |
| APOC3 | IGHV3-13 | LGALS7 |  |
| FBLN1 | IGHV3-7 | HDGF |  |
| CFHR1 | IGHV3-9 | LUM |  |
| UBC | PIGR | APOC4 |  |
| CFB | IGKC | PLTP |  |
| ALB | IGHM | ACTB |  |
| ITIH4 | HBD | LYZ |  |
| PTMA | APOA1 | B2M |  |
| C1S | APOE | TMSB4X |  |
| DAG1 | APOA2 | ACTG2 |  |
| PPIA | FGA | HIST1H4A |  |
| FGG | FGB | TUBA1A |  |
| APOD | CRP | HBB |  |
| CD163 | APCS | HBG2 |  |
| FGG | C1QA | HBA1 |  |
| HGFAC | C1QC | GPLD1 |  |
| GC | C9 | IGLV3-9 |  |
| PROC | APOH | MUC5AC |  |
| THBS4 | LRG1 | ITIH3 |  |
| CFI | FN1 | LGALS3BP |  |
| NRP1 | RBP4 | FGL1 |  |
| CFP | AMBP | SPP2 |  |
| TNC | ORM1 | MMRN1 |  |
| CP | AHSG | APOF |  |
| FETUB | PPBP | SPARCL1 |  |
| SLC35C1 | PF4;PF4V1 | HABP2 |  |
| C8B | TF | FCN2 |  |
