# Supplementary material for: Plasma Proteomic Signatures of Pediatric Sepsis Reveal Persistent Inflammation and Phase‐Specific Biomarkers
Source: FASEB Bioadv. 2026 Mar 9;8(3):e70098. doi: 10.1096/fba.2026-00006 (PMC12972194; doi:10.1096/fba.2026-00006)
Supplement: Supplementary file 3 — Table S2: Uniquely and shared upregulated/downregulated proteins in AP vs. Ctrl and RP vs. Ctrl comparisons. [file FBA2-8-e70098-s002.docx]

**Suppl. Table 2. Uniquely and shared upregulated/downregulated proteins in AP vs Ctrl and RP vs Ctrl comparisons**

| **AP.vs.Ctrl.up** | **RP.vs.Ctrl.up** | **APvs.Ctrl.up\|RPvs.Ctrl.up** | **APvs.Ctrl.down** | **RPvs.Ctrl.down** | **APvs.Ctrl.down\|RP.vs.Ctrl.down** |
| --- | --- | --- | --- | --- | --- |
| SAA2 | LPA | SAA1 | APOF | FCN3 | APOM |
| HPR | C1RL | CRP | RBP4 | SERPINC1 | APOA4 |
| APCS | FCN2 | HP | APOC1 |  | APOA1 |
| CD14 | MST1 | LRG1 | ITIH2 |  | APOA2 |
| APOE | C5 | VWF | SERPIND1 |  | AHSG |
| LCP1 | AZGP1 | LBP | PLG |  | AFM |
| ITIH4 |  | C9 | IGFALS |  | PON1 |
| F10 |  | F9 | KNG1 |  | KLKB1 |
|  |  | ORM1 |  |  | LUM |
|  |  | ITIH3 |  |  | F13A1 |
|  |  | CFB |  |  | A2M |
|  |  | C1R |  |  |  |
|  |  | SERPING1 |  |  |  |
|  |  | C1S |  |  |  |
